# Arsenic trioxide could promote SARS-CoV-2 NSP12 protein degradation

**DOI:** 10.1099/jgv.0.002121

**Published:** 2025-07-07

**Authors:** Tao Yang, Chen Ying Zhu, Pei Han Yu, Chang Yang, Hua Naranmandura

**Affiliations:** 1 Department of Public Health, Zhejiang University School of Medicine, Hangzhou, 310058, PR China; 2 Department of Pharmacology, Zhejiang University School of Medicine, Hangzhou 310058, PR China

**Keywords:** arsenic trioxide, degradation, non-structural protein 12, severe acute respiratory syndrome coronavirus 2 (SARS-CoV-2)

## Abstract

The global dissemination and infection of severe acute respiratory syndrome coronavirus 2 (SARS-CoV-2) have become a worldwide crisis with staggering confirmed cases and death tolls. Although prophylactic vaccines are widely applied to curb the spread of the virus, these protections are greatly weakened by the emergence of SARS-CoV-2 variants. Non-structural protein 12 (NSP12) of SARS-CoV-2 is an RNA-dependent RNA polymerase that plays an essential role in viral replication and transcription, representing a promising target for drug development. Currently, extensive drugs are designed to specifically target and inhibit NSP12 activity, while highly infectious and drug-resistant variants have significantly compromised their efficacy. Here, we identified that arsenic trioxide (ATO) could specifically reduce not only WT SARS-CoV-2 NSP12 but also mutant NSP12 levels, along with low toxicity. Moreover, the reduction of NSP12 was caused by its robust ubiquitination and subsequent degradation via the ubiquitin-proteasome pathway after ATO treatment. Of note, STIP1 homology and U-box containing protein 1 was found to be the E3 ligase responsible for the ubiquitination and degradation of NSP12 by ATO. In short, our findings provide a potential intervention to restrict virus replication and may broaden the scope of therapeutic application for ATO.

## Introduction

Coronavirus disease 2019 (COVID-19) caused by the severe acute respiratory syndrome coronavirus-2 (SARS-CoV-2) has posed a significant threat to public health since the end of 2019 [1,2]. Although various viral protein-targeted drugs, neutralizing antibodies and vaccines are now available in the clinic, the emergence of highly infectious SARS-CoV-2 variants such as omicron has rendered many of them only partially effective and led to multiple waves of infections in the world [3,5].

SARS-CoV-2 has a large genome around 30 kb which contains 14 ORFs encoding 4 structural proteins, 16 non-structural proteins (NSP1-16) and other accessory factors [6,7]. These non-structural and structural proteins play essential roles in the virus life cycle, including viral RNA replication and transcription and virion assembly, as well as inhibition of host immune response [8,9]. Among them, NSP12, the core component of the viral replication–transcription complex, is an RNA-dependent RNA polymerase that plays an important role in catalysing the replication of viral RNA and transcription of the subgenomic RNAs [10,11]. Therefore, it represents a promising target for inhibition of virus replication. Up to now, various NSP12-targeted drugs such as remdesivir have been developed and tested in an effort for the treatment of SARS-CoV-2 infection [12,13]. However, the rapid and unpredictable virus mutation rates lead to the emergence of variants capable of causing breakthrough infections even in fully vaccinated individuals. Thus, it is of paramount importance to explore potential targets and develop novel drugs against variants [14].

Notably, inducing degradation of target proteins by small molecules is an emerging therapeutic modality for elimination of disease-causing proteins through cellular protein degradation pathways [15,16]. Arsenic trioxide (ATO) is one of the most effective targeted drugs for treatment of acute promyelocytic leukaemia, which could selectively target the chimeric oncoprotein PML/RAR*α* (P/R), inducing its SUMOylation as well as ubiquitination, followed by degradation via the ubiquitin-proteasome system, eventually curing the disease [17,18]. Likewise, a recent study has also reported that ATO is capable of targeting the heat shock protein 60 (Hsp60), which triggers the disruption of Hsp60-p53 and Hsp60-survivin complexes, resulting in degradation of p53 mutants and survivin, leading to inhibition of cell proliferation and induction of apoptosis in leukaemia cells [19].

In light of the degradation effect of ATO on disease-causing proteins, we also attempt to explore whether ATO could degrade certain functional proteins of viruses. Here, we found that ATO is able to degrade SARS-CoV-2 NSP12 selectively rather than NSP7/8 and nucleocapsid protein (N protein), implying that ATO may have therapeutic potential for COVID-19 by targeting NSP12 protein.

## Results and discussion

**Fig. 1.**
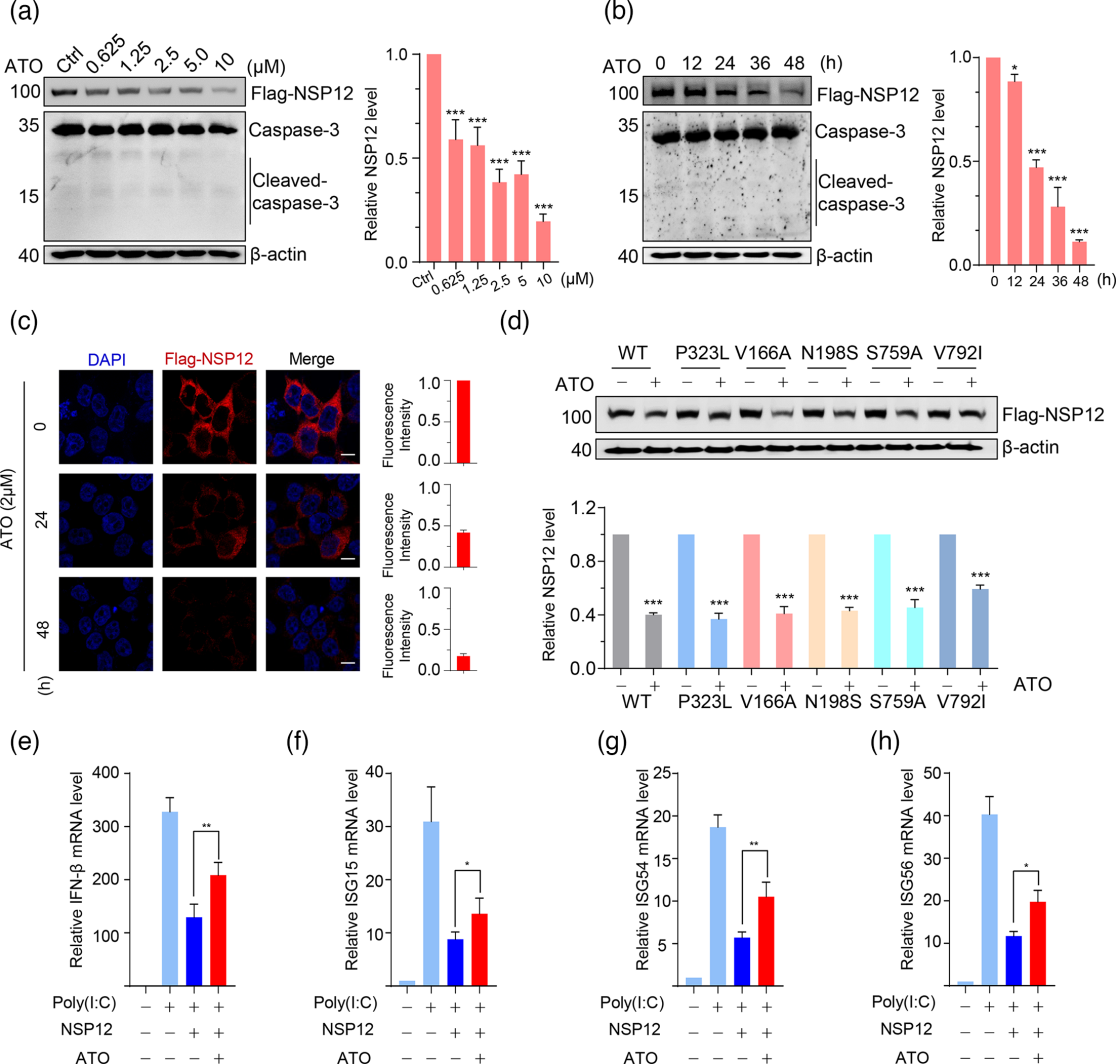
ATO selectively promotes the reduction of SARS-CoV-2 NSP12 level. (**a**) Changes of SARS-CoV-2 NSP12 protein level in 293 T cells after exposure to indicated concentrations of ATO for 24 h were determined by western blot. (**b**) Changes of SARS-CoV-2 NSP12 protein level in 293 T cells after exposure to 2 µM ATO for indicated times were determined by western blot. (**c**) Confocal microscopy analysis for NSP12 level in NSP12 stable-expressed 293 T cells after treatment of 2 µM ATO for 24 and 48 h. The relative fluorescence intensity of each cell was determined by ImageJ and normalized to the control group; data shown are mean±sd (*n*=15). Scale bar in image is 10 µm. (**d**) Western blot analysis for WT and NSP12 mutants in 293 T cells after exposure to 2 µM ATO for 24 h. Quantitative Real Time PCR validation of mRNA expression levels (normalized to GAPDH) for (**e**) IFN-*β*, (**f**) ISG15, (**g**) ISG54 and (**h**) ISG56 in 293 T cells expressing NSP12 following ATO treatment (2 µM, 36 h). Data are mean±sd (*n*=3). Quantitative data of WB are shown as mean±sd (*n*=3). Statistically significant differences of treatment group(s) to the corresponding control group were determined by one-way ANOVA analysis. *P<0.05; **P<0.01; ***P<0.001.

To determine whether the viral proteins of SARS-CoV-2 are sensitive to ATO, 293 T cells that exogenously expressed SARS-CoV-2 N protein, NSP7, NSP8 and NSP12 individually were exposed to ATO. Surprisingly, ATO can specifically reduce the protein level of NSP12 in a time- and dose-dependent manner but has no effect on the N protein and NSP7/8 (Figs 1a, b and S1A–C, available in the online Supplementary Material). Moreover, ATO is also able to specifically reduce NSP12 protein levels when it forms a complex with NSP7 and NSP8 (Fig. S1D). Furthermore, immunofluorescence analysis revealed that NSP12 level was remarkably reduced by ATO treatment (Fig. 1c). In order to clarify that NSP12 protein reduction is specially targeted by ATO but not caused by cell death, the apoptosis-associated protein expression and cell viability were determined. Expectedly, no significant cleaved caspase-3 was observed after ATO treatment, indicating the NSP12 protein degradation is not due to cellular apoptosis (Figs 1a, b and S1A–C). Trypan blue assay and flow cytometry analysis showed that ATO has no significant effects on cell viability at the concentration of 5 µM, while the toxicity became apparent at 10 µM (Fig. S2). All these results demonstrate that ATO could specifically decrease the protein level of SARS-CoV-2 NSP12.

The phenomena of drug resistance and immune evasion of SARS-CoV-2 are typically linked to mutations occurring in critical proteins. The effect of ATO on mutant NSP12 proteins, including the P323L mutant in the Omicron variant and remdesivir-resistant mutants (e.g. V166A, N191S, S759A and V792I), has also been studied by exogenously expressing in 293 T cells [20]. Interestingly, all mutant NSP12 proteins have shown similar sensitivity to ATO treatment as WT NSP12 (Fig. 1d). NSP12 has been shown to antagonize IFN activation. Here, we found that NSP12 suppresses IFN-related gene expression in HEK293T cells, and this suppression is reversed by ATO treatment (Figs 1e–h and S3). These findings confirmed that ATO could selectively reduce levels of WT and mutant NSP12 proteins as well as inhibit NSP12 activity along with low cytotoxicity, providing a potential strategy for the treatment of COVID-19.

**Fig. 2.**
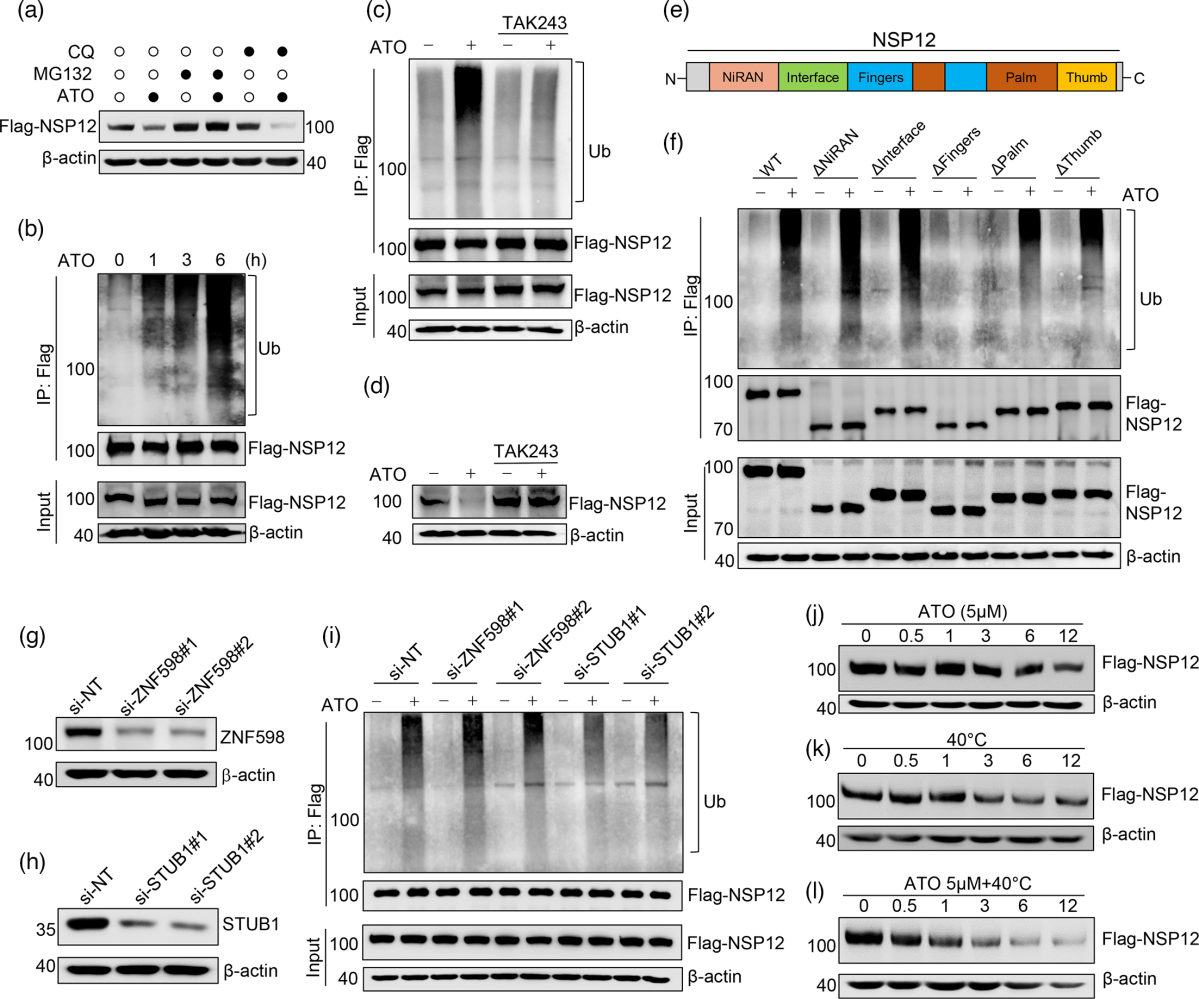
Degradation of SARS-CoV-2 NSP12 by ATO through ubiquitin-proteasome pathway. (a) Flag-NSP12 stable-expressed 293 T cells were pretreated with or without 10 µM MG132 (a proteasome inhibitor) or 20 µM CQ (a lysosome inhibitor) for 1 h and then subjected to ATO treatment (5 µM, 12 h), while the changes in NSP12 protein levels were determined by western blot. (b) Change in ubiquitination level of NSP12 in Flag-NSP12 stable-expressed 293 T cells after exposure to 5 µM ATO for indicated times was determined by immunoprecipitation (IP) with anti-Flag antibody. (c) Flag-NSP12 stable-expressed 293 T cells were pretreated with 1 µM TAK243 (an inhibitor of ubiquitin-activating enzyme) for 2 h followed by ATO treatment (5 µM, 6 h). The change of NSP12 ubiquitination was determined by IP with anti-Flag antibody. (d) Degradation of NSP12 protein level was further determined by western blot in Flag-NSP12 stable-expressed 293 T cells after pretreating with 1 µM TAK243 for 2 h followed by ATO treatment (5 µM, 12 h). (e) Schematic representation of SARS-CoV-2 NSP12 structure with functional domains including NiRAN, Interface, Fingers, Palm and Thumb. (f) WT and NSP12 truncated mutants (including deletion of NiRAN, Interface, Fingers, Palm and Thumb) stable-expressed 293 T cells were treated with ATO (5 µM, 6 h), while the changes in ubiquitination of NSP12 protein were determined by IP with anti-Flag antibody. In addition, 293 T cells were transfected with control siRNA or separate siRNA oligos to knock down E3 ubiquitin ligases ZNF598 and STUB1. Efficiency for knocking down (g) ZNF598 and (h) STUB1 protein levels was analysed by western blot, while the changes in ubiquitination of NSP12 proteins were determined by IP with anti-Flag antibody after knocking down (i) ZNF598 and STUB1. On the other hand, degradation of NSP12 in Flag-NSP12 stable-expressed 293 T cells by (j) ATO, (k) hyperthermia and (l) combination of ATO with hyperthermia was determined by western blot.

Next, we explore the mechanisms involved in ATO-mediated reduction of NSP12 levels. Notably, alterations in protein levels are commonly attributed to changes in transcription levels or protein degradation. As a result, the mRNA level of NSP12 was not changed by ATO treatment (Fig. S4), indicating that the reduction of NSP12 level is not affected by transcription level. Therefore, we assumed that the reduction of NSP12 level might be caused by protein degradation. Generally, ubiquitin-proteasome and autophagy-lysosome systems are two major pathways for mediating protein degradation. Interestingly, the proteasome inhibitor MG132 rather than the lysosome inhibitor chloroquine (CQ) suppressed the ATO-mediated reduction of NSP12 protein (Fig. 2a). Ubiquitination is an essential post-translational modification required for proteasomal degradation. As anticipated, NSP12 protein was indeed robustly ubiquitinated by ATO after several hours (Fig. 2b). Furthermore, pretreatment with TAK243, a ubiquitin-activating enzyme E1 inhibitor, completely blocked NSP12 ubiquitination and subsequent degradation upon ATO treatment (Fig. 2c, d). All these findings demonstrated that ATO-induced NSP12 reduction is owing to protein degradation through the ubiquitin-proteasome pathway.

On the other hand, the NSP12 protein comprises five domains, as shown in Fig. 2(e). When the fingers domain of NSP12 was deleted, ATO-induced protein ubiquitination was completely abolished, indicating that this region is responsive to ATO-induced ubiquitination of NSP12 (Fig. 2f). Moreover, E3 ubiquitin ligase is known as a key enzyme catalysing the ubiquitination process through specifically binding with substrate protein. Therefore, we further explore which E3 ligase participated in ATO-mediated NSP12 ubiquitination. In light of our previous published work, we have already proved that zinc finger protein 598 (ZNF598) is the key E3 ligase for hyperthermia-mediated NSP12 ubiquitination [21]. However, in this study, we identified that STIP1 homology and U-box containing protein 1 (STUB1), a chaperone‐dependent E3 ligase, is the key E3 ligase for ubiquitination of NSP12 protein because knockdown of STUB1 significantly suppressed ATO-mediated NSP12 ubiquitination (Fig. 2g–i). Due to the different mechanisms between arsenic and hyperthermia, we next explore whether these two approaches have a synergistic effect on NSP12 degradation. Surprisingly, the combination of ATO with hyperthermia accelerated the degradation of NSP12 protein (Fig. 2j–l), indicating these two approaches have a synergistic effect on the degradation of NSP12 protein and show potential value in clinical application for COVID-19.

Up to now, several drugs have been investigated for the treatment of COVID-19 through targeting the NSP12 protein of SARS-CoV-2. Among these drugs, remdesivir stands out as the first FDA-approved nucleotide inhibitor specifically aimed at SARS-CoV-2 NSP12. Remdesivir exerts its antiviral effect by incorporating into the template RNA, which effectively halts the elongation of RNA chains, leading to the inhibition of viral RNA synthesis. Favipiravir, a pyrazine derivative, represents another class of nucleotide inhibitors targeting NSP12. Unlike remdesivir, favipiravir impairs RNA extension by inducing mutations within the nascent RNA strand with fewer side effects, potentially making it a more favourable option for clinical treatment. However, the mutations occurring in the NSP12 protein would hinder the antiviral effect of these inhibitors. Herein, we reported for the first time that ATO could also specifically degrade SARS-CoV-2 NSP12 proteins, including WT and mutants, which provide an alternative approach for overcoming drug resistance. In addition, we demonstrated that the mechanism for NSP12 degradation by ATO is predominantly through the following two steps: (1) ATO promotes the ubiquitination of NSP12 through the E3 ligase STUB1 binding with the Fingers domain; (2) the ubiquitinated NSP12 is subsequently degraded through the ubiquitin-proteasome pathway (Fig. 2). Collectively, our results identified that ATO is capable of targeting NSP12 protein, which provides new perspectives for ATO application in virus therapy. Unfortunately, we did not confirm its antiviral activity in the P3 laboratory due to some political limitations, which need to be further investigated in a future study. Metallodrugs have been shown to play a significant role in the prevention and treatment of COVID-19 through directly inhibiting virus replication, preventing virus entry into cells or inhibiting critical enzyme activity. For example, the bismuth compound ranitidine bismuth citrate has demonstrated efficacy in inhibiting the ATPase and DNA-unwinding activities of the SARS-CoV-2 helicase via an irreversible displacement of zinc(II) ions. In contrast to previous studies, our research provides novel evidence that metallodrugs exhibit antiviral effects by degrading viral proteins, thereby offering new insights into the development of antiviral metallodrugs.

## Supplementary material

10.1099/jgv.0.002121 Uncited Supplementary Material 1.

## Abbreviations

ATO: arsenic trioxide
COVID-19: coronavirus disease 2019
CQ: chloroquine
Hsp60: heat shock protein 60
IP: immunoprecipitation
NSP12: non-structural protein 12
SARS-CoV-2: severe acute respiratory syndrome coronavirus 2
STUB1: STIP1 homology and U-box containing protein 1
ZNF598: zinc finger protein 598
