# Supplementary material for: Arsenic trioxide could promote SARS-CoV-2 NSP12 protein degradation
Source: J Gen Virol. 2025 Jul 7;106(7):002121. doi: 10.1099/jgv.0.002121 (PMC12451633; doi:10.1099/jgv.0.002121)
Supplement: Uncited Supplementary Material 1. [file jgv-106-02121-s001.pdf]

Arsenic Trioxide Could Promote SARS-CoV-2 NSP12 Protein Degradation

Tao Yang[a, b†], Chen Ying Zhu[a†], Pei Han Yu[a], Chang Yang[a*], Hua Naranmandura[a,b*]

[a]Department of Public Health and Department of Hematology of First Affiliated Hospital, Zhejiang University School of Medicine, Hangzhou, 310058, China; [b]Department of Pharmacology, School of Medicine, Zhejiang University, Hangzhou 310058, China

Supporting Materials includes Materials and Methods, Supplementary Figure Legends, Supplementary Figures (1-4) and Supplementary Table (1)

Materials and methods

Cell lines and primary cultures. The 293T cell line was obtained from the Cell Bank of the Chinese Academy of Sciences. Upon arrival, the cells were cultured and preserved as a seed stock. Authentication of the cell lines was performed using DNA fingerprinting to ensure no cross-contamination occurred throughout the study. Additionally, an annual check for mycoplasma contamination was conducted. The cells were cultured in DMEM (Gibco, 12800-082) supplemented with 10% fetal bovine serum (Gibco, 10270-106), penicillin (100 U/mL), and streptomycin (100 μg/mL), and maintained at 37°C with 5% CO2.

Reagents and antibodies. Chemicals including chloroquine (S4157), MG132 (S2619) and TAK243 (S8341) were purchased from Selleck. Dylight 549 anti-mouse IgG (A23310) antibody was purchased from Abbkine. STUB1 (68407) and RFP (67378) antibody were obtained from ProteinTech group. Flag antibody (F1804) was purchased from Sigma. GFP (AG281), Ubiquitin (A15546) and HRP labeled anti-mouse (A0216) as well as anti-rabbit (A0208) secondary antibodies were purchased from Shanghai Beyotime biotechnology. β-Actin (AC026) and ZNF598 antibodies were purchased from ABclonal.

Trypan blue cell viability assay. Prior to the assay, cells were treated as specified. After trypsinization and centrifugation, cells were resuspended in PBS and mixed with 0.4% trypan blue. The mixture was incubated for 1 min and then observed under a microscope for viability assessment within three minutes.

Flow Cytometry Analysis. The Annexin V-FITC/PI Apoptosis Kit (Multi Sciences, AP101C) was used for apoptosis assays. Cells were centrifuged, washed twice with PBS solution, and 1-10×10^5 cells were incubated with 5 μL Annexin V-FITC and 10 μL PI at 37°C for 5 minutes. Samples were acquired by on a DxFLEX Flow Cytometer (Beckman Coulter).

RNA Extraction and qPCR Analysis. Total RNA was isolated using TRIzol reagent (Life Technologies) following the manufacturer's protocol. Reverse transcription was performed with 1 μg of RNA using the PrimeScript RT reagent kit (Takara, RR037A). qPCR was carried out with the SYBR green qPCR kit (Takara, DRR820A) on a Bio-Rad CFX-96 system to measure mRNA levels. Gene-specific primer sequences (5'-3') were listed in Supplementary Table 1.

Protein expression and gene silencing. The SARS-CoV-2 NSP12 expression vector was acquired from Hunan Fenghui Biotechnology, while NSP7 and NSP8 vectors were provided by Prof. H. Eric Xu. The N protein plasmid was a gift from Prof. Pei-Hui Wang. Transient and stable expressions were achieved using tagged PCMV and pCDH vectors, respectively. Gene silencing was conducted via siRNA transfection with lipofectamine 3000 (Invitrogen). After transfection for 24h, the cells were treated as indicated and subjected to various analyses. The target sequence of ZNF598 siRNAs

were: si-ZNF598 (#1, CAGGACTACTACAGCGACTAT; #2, GACAATGATGAGCTGCTTAAG), si-STUB1 (#1, CCCAAGTTCTGCTGTTGGACT; #2, GAAGAG GAAGAAGCGAGACAT).

Protein extraction and western blot analysis. Cells were lysed using 8M urea and 0.2mM PMSF-containing RIPA buffer (Beyotime Biotechnology, P0013D). The lysates were chilled on ice for 30 minutes, vortexed every 10 minutes, and then centrifuged at 4°C and 13,000 rpm for 15 minutes to separate the supernatant for subsequent western blotting. Protein concentrations were determined with a BCA kit (Yeasen Biotech, 20201ES90). Protein samples (25 μg) were separated on 7.5-12% SDS-PAGE gels and transferred to PVDF membranes. The membranes were then blocked with non-fat milk and probed with various primary antibodies overnight at 4°C, followed by a 1-hour incubation with HRP-conjugated secondary antibodies at room temperature. Protein bands were detected using enhanced chemiluminescence (Biological Industries, 20-500-120).

Immunoprecipitation and proteomics analysis. $1*10^7$ Cells were lysed in IP buffer (50mM Tris-HCl pH7.5, 10% glycerol, 150 mM NaCl, 2 mM EDTA, 0.5% NP-40, 1 mM PMSF plus protease inhibitors) and immunoprecipitated using Protein A/G PLUS-Agarose Immunoprecipitation Reagent (CST, sc-2003) according to the manufacturer's instructions. The immunoprecipitated proteins were analyzed by western blot.

Immunofluorescence microscopy. Cells were cultured on plates or wells with glass disks and transferred to glass slides after treatments. The slides were then processed by washing with PBS, fixation in 4% paraformaldehyde, and permeabilization with 0.1% Triton X-100. Blocking was performed using 2% BSA in PBS before incubating with primary antibodies at 4°C overnight. The following day, slides were subjected to three PBS washes and incubated with fluorescently tagged secondary antibodies for four hours at room temperature, followed by another three washes. Finally, slides were mounted with DAPI Fluoromount-G and kept in the dark at 4°C until visualization. Fluorescent signals were examined using a Zeiss 510 confocal microscope.

Statistical Analysis. Experiments were replicated at least three times. Statistical significance was determined by unpaired t-test and post-test using Sigmaplot, with $p<0.05$ considered significant (*), $p<0.01$ (**), and $p<0.001$ (***).

Figure Legends

Fig.S1 Effect of ATO on Protein Levels of NSP7, NSP8 and N proteins

(A) GFP-NSP7, **(B)** RFP-NSP8 and **(C)** Flag-N protein transfected 293T cells were treated with indicated concentrations of ATO for 24h, changes in protein levels were determined by western blot. **(D)** 293T cells co-expressing GFP-NSP7, RFP-NSP8 and Flag-N protein were treated with ATO (5μM) for 12h, changes in protein levels were determined by Immunoprecipitation.

Fig.S2 Effect of ATO on Cell Viability

Flag-NSP12 stably expressing HEK293T cells were treated with indicated concentrations of ATO for 24h, **(A)** cell viability was determined by trypan blue assay and **(B)** cell death was determined by flow cytometry analysis. Statistically significant differences of treatment group(s) to the corresponding control group were determined by one-way ANOVA analysis. *$p < 0.05$; **$p < 0.01$; ***$p < 0.001$.

Fig.S3 Effect of ATO on mRNA Level of NSP12

Quantitative RT-PCR validation of mRNA expression levels (normalized to GAPDH) for **(A)** MX1, **(B)** CCL5, and **(C)** CXCL10, in HEK293T cells expressing NSP12 following ATO treatment (2μM, 36h), Data are mean ± SD (n=3). Statistically significant differences of treatment group(s) to the corresponding control group were determined by one-way ANOVA analysis. *$p < 0.05$; **$p < 0.01$; ***$p < 0.001$.

Fig.S4 Effect of ATO on mRNA Level of NSP12

Flag-NSP12 stable expressing 293T cells were treated with indicated concentrations of ATO for 24h, relative level of NSP12 mRNA was detected by RT-qPCR. Data are shown as mean and standard deviation (SD), n=3. Statistically significant differences of treatment group(s) to the corresponding control group were determined by one-way ANOVA analysis. *$p < 0.05$; **$p < 0.01$; ***$p < 0.001$.

Fig.S1

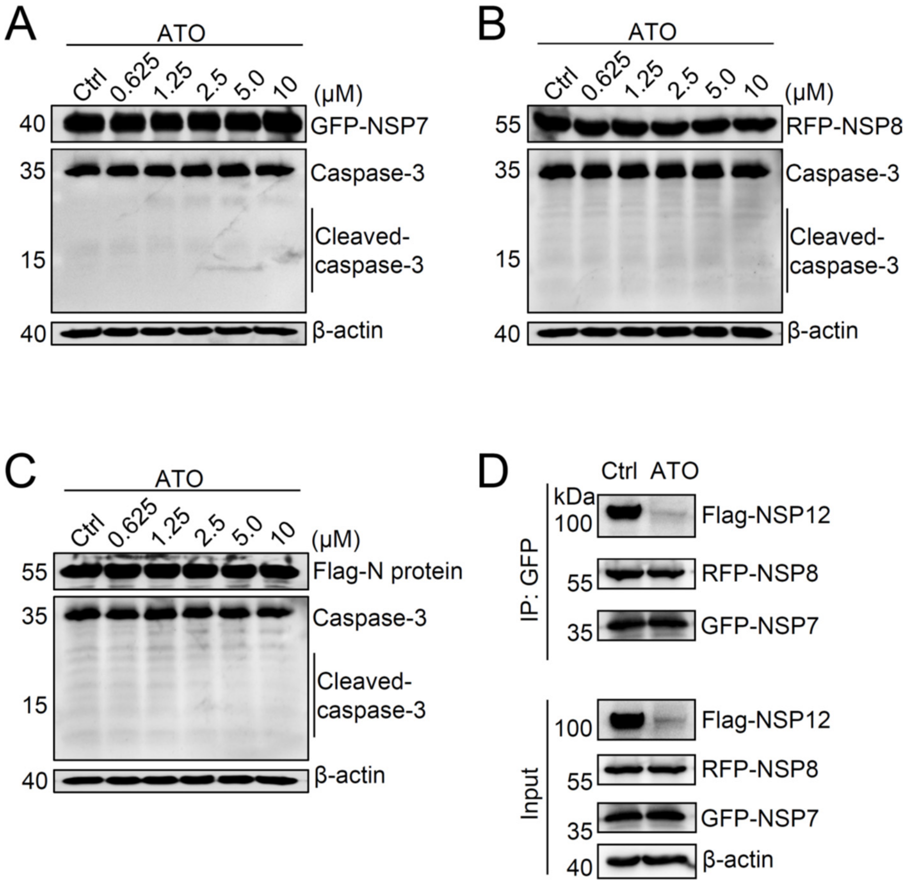
A
ATO
Ctrl
0.625
1.25
2.5
5.0
10
(μM)
40
GFP-NSP7
35
Caspase-3
15
Cleaved-
caspase-3
40
β-actin
B
ATO
Ctrl
0.625
1.25
2.5
5.0
10
(μM)
55
RFP-NSP8
35
Caspase-3
15
Cleaved-
caspase-3
40
β-actin
C
ATO
Ctrl
0.625
1.25
2.5
5.0
10
(μM)
55
Flag-N protein
35
Caspase-3
15
Cleaved-
caspase-3
40
β-actin
D
Ctrl
ATO
kDa
IP: GFP
100
Flag-NSP12
55
RFP-NSP8
35
GFP-NSP7
Input
100
Flag-NSP12
55
RFP-NSP8
35
GFP-NSP7
40
β-actin

Fig.S2

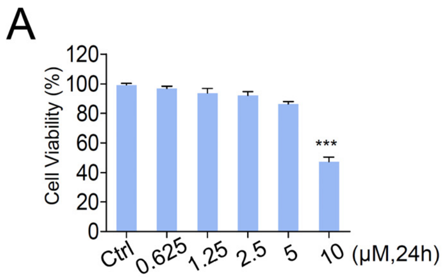
A
Cell Viability (%)
120
100
80
60
40
20
0
***
Ctrl
0.625
1.25
2.5
5
10
(μM,24h)

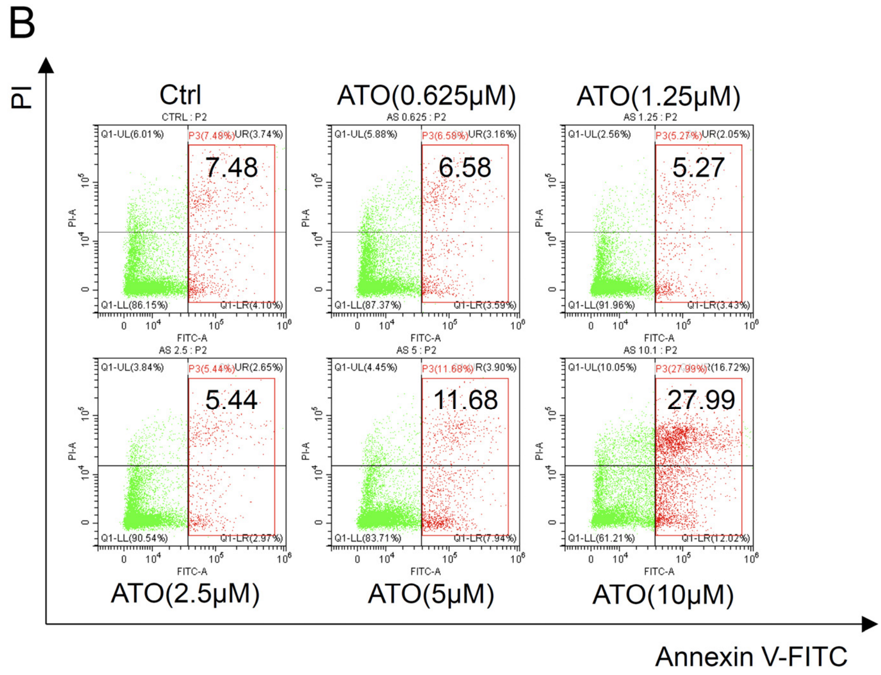
B
PI
Ctrl
ATO(0.625μM)
ATO(1.25μM)
7.48
6.58
5.27
5.44
11.68
27.99
ATO(2.5μM)
ATO(5μM)
ATO(10μM)
Annexin V-FITC

Fig.S3

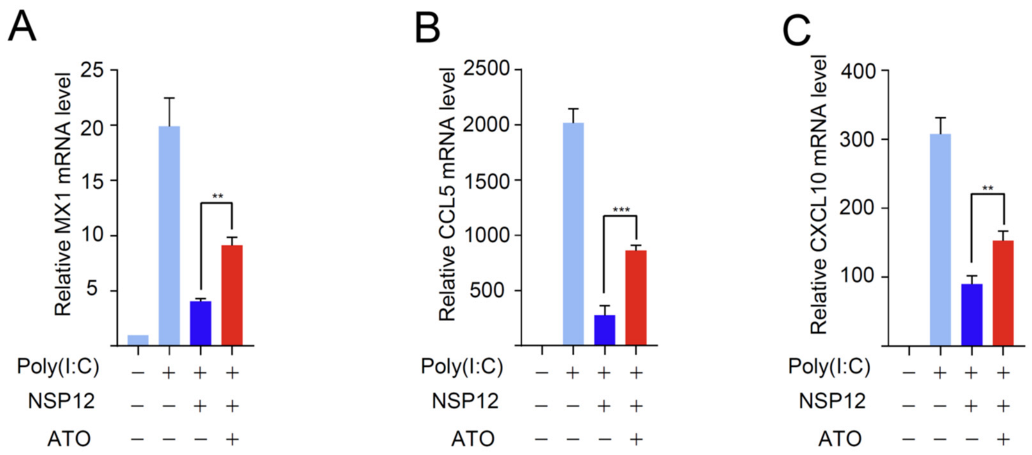
A
Relative MX1 mRNA level
25
20
15
10
5
**
B
Relative CCL5 mRNA level
2500
2000
1500
1000
500
***
C
Relative CXCL10 mRNA level
400
300
200
100
**
Poly(I:C) – + + +
NSP12 – – + +
ATO – – – +

Fig.S4

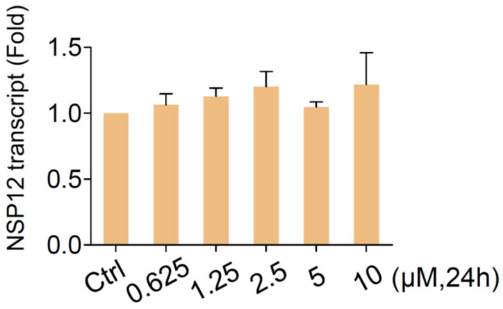

Table S1. Primers used for qPCR Analysis

Transcripts	Forward (5'-3')	Reverse (5'-3')
NSP12	CCGACTTGGTCTATGCCCTC	GCTCGCCGAGATTAGCGTAT
GAPDH	AATCCCATCACCATCTTCCA	TGGACTCCACGACGTACTCA
IFN-β	TAGCACTGGCTGGAATGAG	GTTTCGGAGGTAACCTGTAAG
ISG15	GCGAACTCATCTTTGCCAGTA	AGCATCTTCACCGTCAGGTC
ISG54	ACGCATTTGAGGTCATCAGGG TG	CAGTCGAGGTTATTTGGATTTG GTT
ISG56	TCTCAGAGGAGCCTGGCTAA	TGACATCTCAATTGCTCCAG
MX1	GGTGGTGGTCCCCAGTAATG	ACCACGTCCACAACCTTGTCT
CCL5	CCTGCTGCTTTGCCTACATTGC	ACACACTTGGCGGTTCTTTCGG
CXCL10	GTGGCATTCAAGGAGTACCTC	GACCTTTCCTTGCTAACTGCT
